# Nitrous oxide may not increase the risk of cancer recurrence after colorectal surgery: a follow-up of a randomized controlled trial

**DOI:** 10.1186/1471-2253-9-1

**Published:** 2009-02-03

**Authors:** Edith Fleischmann, Corinna Marschalek, Katja Schlemitz, Jarrod E Dalton, Thomas Gruenberger, Friedrich Herbst, Andrea Kurz, Daniel I Sessler

**Affiliations:** 1 Department of Anesthesiology and Intensive Care and Pain Medicine, Medical University Vienna, Vienna, Austria; 2 Department of General Surgery, Medical University Vienna, Vienna, Austria; 3 Department of Quantitative Health Sciences, The Cleveland Clinic, Cleveland, Ohio, USA; 4 Outcomes Research, The Cleveland Clinic, Cleveland, Ohio, USA

## Abstract

**Background:**

Even the best cancer surgery is usually associated with minimal residual disease. Whether these remaining malignant cells develop into clinical recurrence is at least partially determined by adequacy of host defense, especially natural killer cell function. Anesthetics impair immune defenses to varying degrees, but nitrous oxide appears to be especially problematic. We therefore tested the hypothesis that colorectal-cancer recurrence risk is augmented by nitrous oxide administration during colorectal surgery.

**Methods:**

We conducted a 4- to 8-year follow-up of 204 patients with colorectal cancer who were randomly assigned to 65% nitrous oxide (n = 97) or nitrogen (n = 107), balanced with isoflurane and remifentanil. The primary outcome was the time to cancer recurrence. Our primary analysis was a multivariable Cox-proportional-hazards regression model that included relevant baseline variables. In addition to treatment group, the model considered patient age, tumor grade, dissemination, adjacent organ invasion, vessel invasion, and the number of nodes involved. The study had 80% power to detect a 56% or greater reduction in recurrence rates (i.e., hazard ratio of 0.44 or less) at the 0.05 significance level.

**Results:**

After adjusting for significant baseline covariables, risk of recurrence did not differ significantly for nitrous oxide and nitrogen, with a hazard ratio estimate (95% CI) of 1.10 (0.66, 1.83), *P *= 0.72. No two-way interactions with the treatment were statistically significant.

**Conclusion:**

Colorectal-cancer recurrence risks were not greatly different in patients who were randomly assigned to 65% nitrous oxide or nitrogen during surgery. Our results may not support avoiding nitrous oxide use to prevent recurrence of colorectal cancer.

**Implications Statement:**

The risk of colorectal cancer recurrence was similar in patients who were randomly assigned to 65% nitrous oxide or nitrogen during colorectal surgery.

**Trial Registration:**

Current Controlled Clinical Trials NCT00781352

## Background

Colon cancer is the third most common cause of cancer in the United States. Surgery is the standard treatment for colorectal cancer and is often curative. Nevertheless, cancer surgery is associated with release of tumor cells into the systemic circulation [1], and it is likely that *minimal residual disease *is present after even the best surgery. Whether disseminated tumor cells are able to establish metastases depends on several factors, including the efficacy of host immune responses, in particular the function of natural killer cells which are the primary defense against malignancy [2,3].

Perioperative factors that impair host immunity are thus likely to facilitate local recurrence or establishment of metastatic tumor after cancer surgery [4]. For example, metastasis formation in murine models is promoted by anesthetic drugs, most of which are immunosuppressive [5-7]. The extent to which various anesthetic drugs depress natural killer cell and other immune functions related to countering malignancy varies considerably [5,7-9]. Nitrous oxide has been given to more than a billion surgical patients and possibly remains the most commonly used general anesthetic. Nonetheless, it is well established that this inhaled anesthetic interacts with vitamin B12, resulting in selective inactivation of methionine synthase that is a key enzyme in methionine and folate metabolism. Nitrous oxide thus impairs one-carbon and methyl-group transfers which are critical for DNA, purine, and thymidylate synthesis [10]. Impaired synthesis restricts formation of new cells such as those of the hematopoietic system [11-13]. Nitrous oxide also depresses neutrophil chemotaxis [14] and reduces proliferation of human peripheral blood mononuclear cells [14].

Among the cells impaired by nitrous oxide are the immune cells that fight malignancy [11,15]. For example, nitrous oxide not only augments metastases in murine experimental models, but generates metastases in organs that are usually resistant [5]. Thus lung metastases in a mouse tumor model were twice as common when major surgery was conducted with nitrous oxide than with sodium thiopental, ketamine, or halothane.

Local or distant recurrence after cancer surgery is thus likely to be determined in part by a body's ability to handle minimal residual disease in the immediate perioperative period. Most anesthetics impair defenses against malignancy, especially natural killer cells, and nitrous oxide may be worse than others. We therefore tested the hypothesis that nitrous oxide administration during colorectal cancer surgery enhances recurrence risk.

## Methods

In a previously published study, we evaluated the effect of nitrous oxide on the incidence of postoperative wound infection after colectomy [16]. Our major conclusion was that nitrous oxide does not increase the incidence of postoperative wound infection after colon resection. The current study evaluated cancer recurrence in those patients whose original indication for surgery was colorectal malignancy. Patients were enrolled between November 11, 1998, and November 3, 2002, and our follow up extended until March 23, 2007; cancer recurrence was thus evaluated over a 4- to 8-year period. The study was not registered because patient enrollment preceded development of major public trial registries.

Both the original and the current study were conducted with the approval of the Institutional Review Boards of the participating hospitals. Written informed consent was obtained for the original study, i.e., nitrous oxide/postoperative wound infection, and then was waived for the current study. Briefly, patients enrolled in the original study were aged 18 to 80 years old. All had colon resections, usually for cancer or inflammatory bowel disease. All patients were given 35% inspired oxygen during surgery, which was balanced by either 65% nitrous oxide or nitrogen that was randomly assigned. The remainder of the isoflurane and remifentanil anesthetic was standardized, as was antibiotic prophylaxis. All patients were kept normothermic because hypothermia triples infection risk [17].

**Figure 1:**
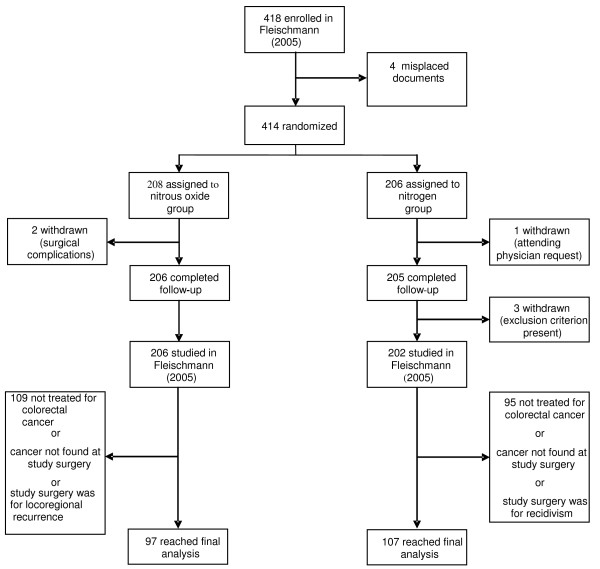
**Trial profile after Fleischmann E, Lenhardt R, Kurz A et al 2005**.

Among the 418 patients enrolled in the initial study, 10 were excluded from analysis for various reasons. 206 patients in the nitrous oxide group and 202 patients in the nitrogen group thus completed the trial. Among these, 97 given nitrous oxide and 107 given nitrogen had initial colorectal cancer surgery and were available for this analysis (fig. 1).

Follow-up was conducted by investigators who were blinded to the original assignment to nitrous oxide or nitrogen. Cancer registries were reviewed, patients were contacted, and their health status confirmed with their oncologists and/or general practitioners. Family members and government authorities were contacted as necessary to determine patients' current addresses or confirm mortality. Our primary outcome was local or metastatic recurrence of colon cancer.

Baseline factors that were evaluated included age, sex, diagnoses, preoperative carcinoembryonic antigen (CEA), neoadjuvant treatment, site of tumor, Dukes and TNM classification, tumor grading, number of examined lymph nodes, number of involved nodes, vessel and neural invasion, dissemination, and whether patients had a history of malignancies other than colorectal cancer. Using survivor function estimates from the control group (nitrogen), with 39/97 observed events in the nitrous-oxide group and 38/107 observed events in the nitrogen group, we had an 80% power to detect a hazard ratios less than 0.44 or greater than 2.3 at the 0.05 significance level, if such hazard ratios indeed existed. Since more subtle effects of nitrous oxide are likely of clinical interest, our study should be considered exploratory.

### Data Analysis

Cancer-free survival time was defined as the time from surgery to recurrence for those patients who experienced recurrence, and the time from surgery to the earlier of the follow-up date and the death date (if something other than cancer caused the death) for those patients who were censored (i.e., did not experience an observed recurrence before being lost to follow-up). Patients who did not have a recorded recurrence date but died of cancer were recorded as having a recurrence date similar to the date of death. Due to lack of follow-up information, one patient was censored on the first postoperative day. Four others were censored within the first 40 postoperative days. All but one other patient had at least 270 days of follow up.

Univariable comparison of the randomized groups for time to recurrence of cancer was performed visually using Kaplan-Meier estimates of the survivor function and statistically using the log-rank test. Cox proportional hazards regression was also used to assess the relationship between baseline factors and recurrence.

A multivariable-Cox-proportional-hazards-regression model including all baseline variables independently significant at *P *< 0.25 (that is, significant at *P *<*0.25 *in the presence of other covariables) in the model, was fit in order to better balance the treatment groups and to increase precision in the estimation of the effect of type of gas on survival. Any factor showing some univariable relationship with the type of gas administered (as determined by univariable tests with a significance criterion of *P *<*0.40*) was considered building the model.

Statistical analysis was performed with SAS statistical software, Cary, NC, and the R programming language, Vienna, Austria.

## Results

Demographic, morphometric, and perioperative characteristics of the treatment groups, are presented in Table 1. Though we did not expect any imbalance in these characteristics between the two groups due to randomized allocation of type of inhaled gas, patients in the nitrous-oxide group had slightly higher preoperative CEA values (median [quartiles] of 4.9 [1.7, 13]) than patients in the nitrogen group (3.4 [1.9, 7.5]). Additionally, patients given nitrous oxide had some combination of chemotherapy and radiation more frequently (61%) than patients given nitrogen (52%). Nonetheless, these differences were not statistically significant (*P *= 0.70 and *P *= 0.30, respectively). Time-weighted end-tidal isoflurane partial pressure was slightly, but significantly, greater in patients assigned to nitrogen: 0.64% *vs*. 0.56% (*P *< 0.001). The fraction of patients transfused and the number of transfused units were similar in each group; opioid use in the first two postoperative hours was also similar.

**Table 1:** Baseline Characteristics for 204 Colorectal Cancer Patients.

		**Nitrous Oxide**	**Nitrogen**	
**Factor**	**Level**	**N = 97**	**N = 107**	***P*-Value**
Gender	Female	40 (41)	39 (37)	0.48
Hospital	AKH	87 (90)	94 (88)	0.68
	SMZ-OST	10 (10)	13 (12)	
Dukes classification	A	17 (19)	16 (16)	0.96
	B	27 (30)	31 (30)	
	C	33 (36)	38 (37)	
	D	14 (15)	17 (17)	
Tumor Grade	G1	3 (3)	5 (5)	0.68
	G2	67 (74)	75 (72)	
	G3	19 (21)	24 (23)	
	G4	1 (1)	0 (0)	
Site	Ascending Colon	10 (11)	18 (17)	0.73
	Sigmoid Colon	28 (30)	30 (28)	
	Rectal	45 (48)	44 (42)	
	Cecum	10 (11)	13 (12)	
	Multiple	1 (1)	1 (1)	
Dissemination	Yes	23 (24)	25 (24)	0.92
Invasion to Adjacent Organs	Yes	31 (33)	28 (26)	0.33
Neural Invasion	Yes	9 (10)	10 (9)	0.95
Vessel Invasion	Yes	21 (23)	27 (26)	0.63
Treatment of Tumor besides surgery	None	36 (39)	50 (48)	0.30
	Chemotherapy	30 (32)	30 (29)	
	Radiation	15 (16)	9 (9)	
	Both	12 (13)	16 (15)	
Age (Years)		61 ± 11	63 ± 11	0.41
Number of Nodes examined		16 [12, 21]	15 [12, 23]	0.97
Number of involved Nodes		0 [0, 4]	1 [0, 3]	0.88
Preoperative CEA		4.9 [1.7, 13.0]	3.5 [1.9, 7.5]	0.70
Recurrence-free survival (years)		3.9 [1.2, 5.4]	4.4 [1.1, 5.2]	0.89

AKH is the Vienna General Hospital; SMZ-OST is the Danube Hospital, also in Vienna, Austria. Statistics presented as number (%), means ± SDs, or medians [first-to-third quartile range]. P-values, respectively, are from Pearson's Chi-Squared Test, Student's T-Test, and Wilcoxon's Rank Sum Test.

Median [quartiles] follow-up time was 4.2 [1.2, 5.4] years. During follow-up, 38% (N = 36) of the nitrous oxide and 37% (N = 40) of the nitrogen patients died. Independent of the available covariables, use of nitrous oxide was associated with an estimated 33% reduction (95% CI -63%, +22%) in mortality rate (*P *= 0.19).

**Figure 2:**
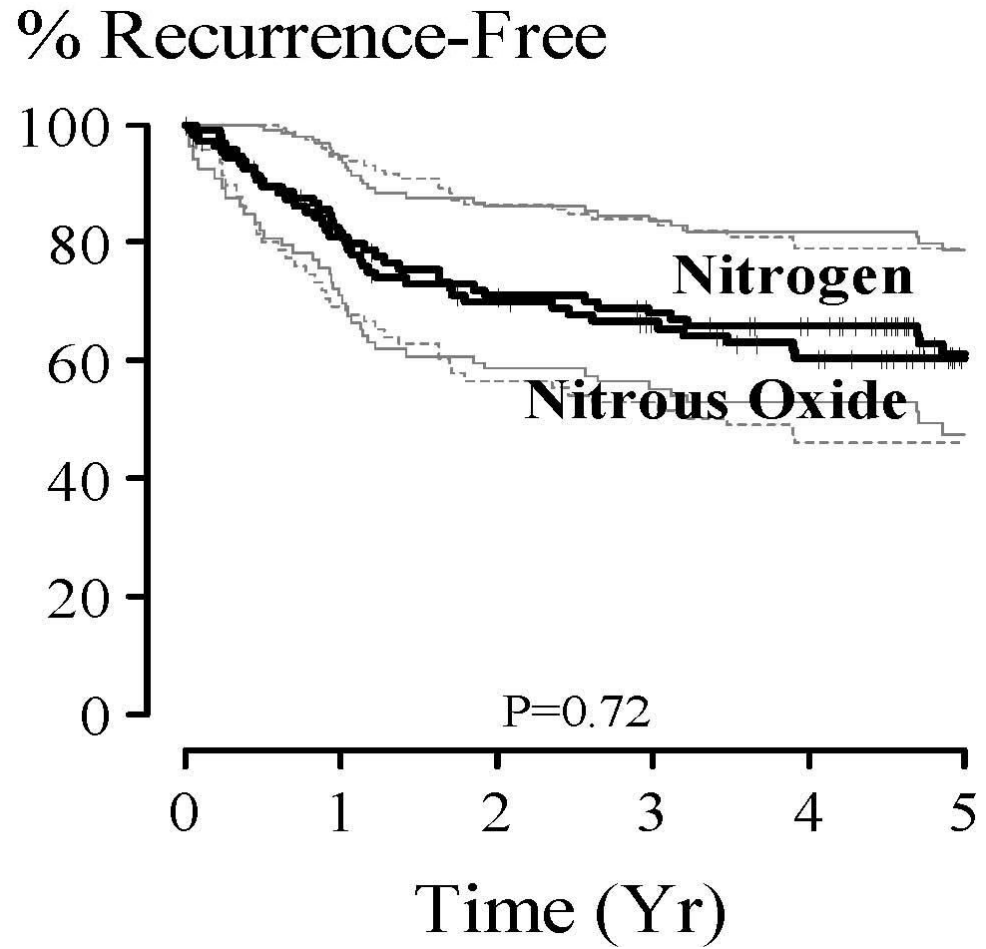
**Kaplan-Meier Estimates (and 95% Equal Precision Confidence Bands) of recurrence-free survival for 107 Colorectal Cancer Patients Receiving Nitrogen Gas (solid confidence bands) and 97 Receiving Nitrous Oxide Gas (dotted confidence bands)**. Multivariable P = 0.72.

On univariable analysis, no difference was found between the nitrous oxide and nitrogen groups on cancer recurrence rates. Specifically, the ratio (95% CI) of cancer recurrence risk (i.e., hazard ratio) at any given point in time (nitrous oxide vs. nitrogen) was estimated at 1.14 (0.73, 1.79), *P *= 0.56. Kaplan-Meier survivor function estimates are summarized in Figure 2 and Table 2. The relationship between baseline factors and survival is given in Table 3; as expected, many factors were predictive of survival at the 0.05 significance level.

**Table 2:** Summary of Kaplan-Meier Survivor Density Function Estimates.

	**Nitrous Oxide N = 97**				**Nitrogen N = 107**			
**Time**	**Survival (95% CI)**	**# Events**	# Censored*	**# Left**	**Survival (95% CI)**	**# Events**	# Censored*	**# Left**
6 Months	89 (83, 96)	10	3	84	90 (85, 96)	10	3	94
1 Year	81 (73, 89)	18	4	75	82 (74, 89)	19	4	84
2 Years	70 (61, 79)	28	6	63	71 (62, 80)	30	5	72
3 Years	67 (57, 76)	31	10	56	68 (59, 77)	33	8	66
5 Years	60 (50, 71)	36	29	32	61 (51, 71)	38	37	32
Last Observation	49 (34, 64)	39	58	0	61 (51, 71)	38	69	0

* Lost to follow-up before observed recurrence.

**Table 3:** Univariable Cox Regression Model Results.

**Variable**	**Category or Units**	**N**	**Hazard Ratio (95% C.I.)**	***P*-Value**
*Gas*	*Nitrogen*	*107*	*1.0*	*0.56*
	*Nitrous Oxide*	*97*	*1.14 (0.73, 1.79)*	
Age	10 Years	204	1.12 (0.91, 1.36)	0.29
Gender	Male	125	1.0	0.29
	Female	79	1.27 (0.81, 2.00)	
Hospital	AKH	181	1.0	0.07
	SMZO	23	0.91 (0.45, 1.83)	
Number of Nodes examined	1 Node	194	1.00 (0.90, 1.11)	0.98
Number of involved Nodes	1 Node	194	1.17 (1.12, 1.22)	< 0.001
Tumor Grade	G1	8	1.0	0.06
	G2	142	1.35 (0.33, 5.58)	
	G3	43	2.60 (0.61, 11.1)	
	G4	1	--	
Treatment of Tumor	None	86	1.0	0.01
	Chemotherapy	60	1.84 (1.08, 3.16)	
	Radiation	24	0.82 (0.33, 2.00)	
	Both	28	2.39 (1.27, 4.50)	
Log_2_(Preoperative CEA)	1 (Doubling)	144	1.32 (1.21, 1.44)	< 0.001
Site	Ascending	28	1.0	0.052
	Sigmoid	58	2.99 (1.24, 7.25)	
	Rectum	89	2.23 (0.94, 5.32)	
	Cecum	23	1.36 (0.41, 4.45)	
	Multiple Colon Cancer	2	--	
Dukes Classification	A	33	1.0	< 0.001
	B	58	1.27 (0.38, 4.21)	
	C	71	5.23 (1.85, 14.7)	
	D	31	16.5 (5.72, 47.7)	
Dissemination	No	153	1.0	< 0.001
	Yes	48	7.82 (4.89, 12.5)	
Adjacent Organ Invasion	No	142	1.0	< 0.001
	Yes	59	2.28 (1.44, 3.61)	
Neural Invasion	No	180	1.0	< 0.001
	Yes	19	3.26 (1.84, 5.77)	
Vessel Invasion	No	151	1.0	< 0.001
	Yes	48	3.37 (2.11, 5.36)	

After adjusting for significant baseline covariables (multivariable model, Table 4), risk of recurrence did not differ significantly for nitrous oxide and nitrogen, with a hazard ratio estimate (95% CI) of 1.10 (0.66, 1.83), *P *= 0.72. The multivariate model included treatment group as well as age, tumor grade, dissemination, adjacent organ invasion, vessel invasion, and number of involved nodes. No two-way interactions with the treatment were statistically significant. A plot of log [-log(survival)] against log(time) showed that the hazard ratio (or risk ratio) of recurrence between the experimental groups did not change over time, satisfying this important assumption of the Cox proportional hazards model.

**Table 4:** Final Multivariable Cox Regression Model Results.

**Model Parameter**	**Reference or Units**	**Hazard Ratio (95% C.I.)**	***P*-Value**
*Nitrous Oxide*	*Nitrogen*	1.10 (0.66, 1.83)	0.72
Age	10 Years	1.16 (0.93, 1.44)	0.18
Tumor Grade	1	0.73 (0.43, 1.25)	0.25
Dissemination (Yes)	No	5.6 (3.1, 10.2)	< 0.001
Invasion into Adjacent Organs (Yes)	No	0.60 (0.30, 1.23)	0.16
Invasion into Vessels (Yes)	No	1.54 (0.87, 2.70)	0.14
# involved Nodes	1 Node	1.16 (1.09, 1.24)	< 0.001*

## Discussion

Despite significant improvements in diagnosis, surgical technique, and adjuvant therapies, cancer recurrence remains the primary cause of death in patients presenting with colonic malignancies. Cancer surgery, sometimes the best hope for cure, is nearly always associated with minimal residual disease [1], and competence of host defense, especially natural killer cell function, appears to be a critical determinant of whether residual disease develops into clinical recurrence [4,18].

Anesthetics impair immune function to various degrees [5,13]; and at least in animals, anesthetic interventions that impair natural killer cells augment metastatic risk [5,7,8,19,20]. For example, recurrence is more common with general than regional anesthesia in retrospective analyses of 129 women with breast cancer [21] and 225 men with prostate cancer (unpublished data). Similar benefits of avoiding general anesthesia has been observed with melanoma surgery [22]. Perioperative blood transfusion, which impairs natural killer cell function [23] and promotes angiogenesis (which is necessary for metastases) [24], may also increase recurrence risk after some types of cancer surgery [25] – although this conclusion remains controversial [26,27].

Nitrous oxide impairs numerous immune functions, including those critical for fighting cancer [5,11,15]. Our study has limited power resulting from its relatively small sample size. Increases in recurrence risk to as much as 83% or decreases by as much as 34% cannot be excluded. A larger, randomized, clinical trial would thus be helpful. We nonetheless note that previous major studies evaluating the effect of anesthetic interventions on cancer-recurrence risk in humans were retrospective; ours differs in being the first prospective randomized trial with blinded outcome assessment which markedly strengthens our conclusion.

In our study, postoperative analgesia was provided by patient-controlled intravenous opioids. In the first two postoperative hours, opioid use was similar in patients assigned to either nitrous oxide or nitrogen. Unfortunately, opioid use beyond the first two hours of recovery was not recorded. While it is thus possible that this confounds our analysis, it seems unlikely that intraoperative administration of nitrous oxide, an extremely short-acting drug, would alter opioid requirements beyond the initial recovery period.

Our original randomized study comparing nitrous oxide to nitrogen had as its major outcome surgical wound infection [16], with bowel distention being a secondary outcome [28]. The primary enrollment criterion for our original study was thus colon resection; however, only about half of our patients had surgery for cancer. The only inclusion criteria for this study, surgery for colorectal cancer, was applied after randomization of patients. However, there was no reason to believe that the anesthetic groups within this chosen subset of patients would not be balanced on baseline characteristics. As might thus be expected, the groups did not differ substantially in terms of baseline risk. Furthermore, our multivariate analysis compensated for present baseline differences.

Of the 77 observed recurrences (per our definition), eight did not have a diagnosed recurrence; instead, their death certificates listed colorectal cancer as the cause of death. Though this assumption relies on the accuracy of death certificate information, we believe it is justifiable to assume they recurred.

The primary anesthetic in our original study was isoflurane, which impairs natural killer cells [8] and other immune functions [20,29]. Patients assigned to nitrous oxide were given 0.56 ± 0.13% isoflurane and those assigned to nitrogen were given 0.64 ± 0.14%. While these concentrations differed significantly, the difference was trivial and almost surely unimportant. A consequence of the original study being designed and powered for infection is that we had limited power to detect an effect of nitrous oxide on recurrence of colorectal cancer. Nonetheless, our results suggest that if nitrous oxide promotes cancer recurrence, the relative risk is modest.

## Conclusion

In summary, colon-cancer recurrence risk was similar in patients randomly assigned to intraoperative nitrous oxide or nitrogen. Our power was relatively low, thus leaving the possibility that nitrous oxide reduces risk by as much as 34% or increases it by as much as 83%. Nonetheless, our results do not support avoiding nitrous oxide use to prevent recurrence of colorectal cancer.

## Competing interests

The authors declare they have no competing interests.

## Authors' contributions

EF designed the study, collected data, and drafted the manuscript. CM collected the data and helped to write the manuscript. KS collected the data. JD performed the statistical analysis. TG and FH participated in the design of the study and helped to interpret the results. AK and DS participated in the design of the study, interpreted the data and drafted the manuscript. All authors read and approved the final manuscript.

## Pre-publication history

The pre-publication history for this paper can be accessed here:


